# Supplementary material for: Dietary Omega-3 PUFAs in Metabolic Disease Research: A Decade of Omics-Enabled Insights (2014–2024)
Source: Nutrients. 2025 May 28;17(11):1836. doi: 10.3390/nu17111836 (PMC12157617; doi:10.3390/nu17111836)
Supplement: Supplementary file 1 [file nutrients-17-01836-s001.zip › nutrients-3593548-supplementary.pdf]

Table S1. Omics research findings in animal models.

Omic Type	Animal	Group/Intervention	Duration	Tissue Evaluated	Main Outcome	Ref.
Transcriptomics, Lipidomics, Proteomics	C57BL/6J mice, fat-1 mice	Wild Type (WT) pups +high Omega-6/Omega-3 PUFAs ratio milk;<br>WT pups+low Omega-6/Omega-3 PUFAs ratio milk;	10 days	Serum and adipose tissue	↑FA oxidation and beigeing of APCs;<br>↑Beige adipocyte mRNAs Ppargc1a, Ucp2, and Runx1 and NR2F2 protein;<br>↓Omega-6/Omega-3 FA ratio;<br>↓Adipogenesis-related factors: ASP, Adipsin, ALS, and RBP4 concentrations;<br>↓White adipose tissue.	[1]
Transcriptomics	Duroc pigs	Conventional diet;<br>Western diet: high fat content and protein of animal origin (caseinate);<br>Western diet with *Bifidobacterium* breve and rice hydrolysate;<br>Western diet with *Bifidobacteriu*m breve and rice hydrolysate+ Omega-3 PUFAs.	10 weeks	Serum, liver, and adipose tissue	↓Body weight gain, fat mass, LDL-cholesterol;<br>↓Adipogenesis and inflammation;<br>↑Fatty acid oxidation and cholesterol catabolism.	[2]
Transcriptomics	C57bl/6J mice	F1 Omega 3 lineage:fish oil, 22% MUFA,19%SFA,59% PUFA(Omega-6:Omega-3 ratio=3.5), 8%EPA-0.3%DHA;<br>Control lineage:sunflower oil, 36%MUFA,19%SFA,45%PUFA(Omega-6:Omega-3 ratio=10);<br>F3 Omega 3 lineage+HFD(Hfepa)(HFD, 24% of fat, 20% of sucrose);<br>F3 control lineage+HFD (Hfoleic);<br>F3 control lineage+LFD (reference).	17 weeks	Visceral adipose tissue (VAT)	↑PI3KAKT-mTOR-signaling pathway;<br>↑Chemokine signaling pathway: Akt and Pi3k genes;<br>↓Foxo, Gsk3 beta;<br>↓Weight, fat mass.	[3]
Transcriptomics	C57BL/6 mice	Chow diet;<br>HFD:20% kcal protein, 20% kcal carbohydrate, and 60% kcal fat;<br>HFD+EPA:20% kcal protein, 20% carbohydrate, 60% kcal fat enriched with 3.2% purified EPA;<br>HFD+DHA:20% kcal protein, 20% carbohydrate, 60% kcal fat enriched with 3.2% purified DHA.	10 weeks	Liver and muscle	↓Insulin tolerance;<br>↓Extracellular matrix in muscle, inflammation.	[4]
Transcriptomics	Iberian×Duroc crossbred pigs	High Omega-6:Omega-3 PUFAs ratio diet;<br>Low Omega-6:Omega-3 PUFAs ratio diet.	/	Longissimus dorsi (LD) skeletal muscle	DEmiRs: ssc-miR-15b, ssc-mir30a-3p, ssc-miR-30e-3p and ssc-miR-7142-3p:adipogenesis and inflammation miRNA-to-mRNA predicted interactions:mir15b-ARRDC3; mir7142-3p-METTL21C: lipolysis, obesity, myogenesis, and protein degradation.	[5]
Transcriptomics, Epigenomics	Sprague–Dawley (SD) rats	Control group (n=8, without fructose in drinking water or DHA supplement);<br>15% Fructose treatment (n=8, 15% w/v fructose in the drinking water);<br>15% Fructose+Omega-3 fatty acid diet rich in DHA (n=8; 0.5% of flaxseed oil supplying ALA and 1.2% of DHA capsule oil).	6 weeks	Hypothalamus and hippocampus	↓Serum triglycerides, insulin, insulin resistance index;<br>↑Hypothalamus: Bgn, Fmod;<br>↓Hippocampus: Bgn, Fmod;<br>DHA reverses biological pathways and gene networks perturbed by fructose via transcriptional regulators (such as transcription factors, epigenetic regulators, splicing factors) and essential network regulators (such as Bgn and Fmod) that may control the balance between health and disease.	[6]

Transcriptomics, Lipidomics	C57BL/6J mice	Control diet;<br>HFD-EPA/DHA;<br>HFD-corn oil.	8,12 weeks	Liver, adipose tissues, and plasma	↓Body weight gain and adiposity,liver triglyceride content;<br>↓Neutral lipids:C16:0 ,C18:0; Phospholipids:C18:0, C18:1n-9, C20:4n-6;<br>↑Neutral lipids:EPA (C20:5n-3) and DHA (C22:6n-3) ; Phospholipids: EPA, DHA, C16:0;<br>↓Lipoprotein particle assembly (ApoA4), fatty acid transport (Fabp2) and biosynthesis of long-chain PUFA (Elovl2, Elovl5, Fads1 and Fads2).	[7]
Transcriptomics	C57BL/6 mice	Calorie restriction (CR):13.6 kcal/day;<br>CR-Lard:8.6 kcal/day, MUFAs and SFAs;<br>CR-Soy:8.6 kcal/day,soybean oil,high in Omega-6 PUFAs, 55% LA;<br>CR-Fish:8.6 kcal/day,fish oil, high in Omega-3 PUFAs, 18% EPA, 12% DHA.	8 months	Liver and the entire hindlimb skeletal muscle	↓Body weight and the weight of most organs;<br>↑Proinflammatory cytokines (TNF, GM-CSF, TGF-β) and sex hormones(β-estradiol). mitochondrial and peroxisomal fatty acid β-oxidation;<br>↑PPARα,TNF,TGF-β1, and leptin signaling;<br>↓Fatty acid biosynthesis SREBP-1,Fasn, Acc1,Fads1 and Fads2.	[8]
Transcriptomics, Lipidomics	C57BL/6J mice	Control diet;<br>HFD-ED:2 % (w/w): EPAX (n-3 enriched) oils (EPA; EPAX 1050 and DHA; EPAX 6015) and 3 % (w/w) corn oil;<br>HFD-Corn Oil:5 % (w/w) corn oil.	8 weeks	Blood and adipose tissue: White and brown adipose	↑C20:5 n-3 (EPA), C22:5 n-3 (DPA), C22:6 n-3 (DHA), SFA (C12:0, C14:0, C16:0);<br>↓n-6 PUFAs, MUFA (C18:1 n-9、C18:1 n-7), lipid accumulation and levels of the pro-inflammatory fatty acid arachidonic acid;<br>↑Pparα,Prdm16;<br>↑Electron transport chain, fatty acid β-oxidation, mitochondrial-translation, and organization;<br>↓Biosynthesis of fatty acid, phospholipid, cardiolipin, cholesterol and sterol.	[9]
Transcriptomics	C57BL/6J mice	F1 Omega-3 lineage:fish oil,75% of Omega-3 FA;<br>Control lineage:sunflower oil,83.5% of oleic acid;<br>F3 Omega-3 lineage+HFD(Hfepa);<br>F3 control lineage+HFD (Hfoleic);<br>F3 control lineage+LFD (reference).	17 weeks	Liver	↓Fat mass accumulation, liver weight, plasma glucose, and insulin levels;<br>↓miR-34a-5p.	[10]
Proteomics, Lipidomics	Israeli-Holstein dairy cows	CTL cows: 250 g/d/cow calcium salt of fatty acids (Adolac, Poliva, Israel) and PP supplemented at 1.6% of the diet (DM basis);<br>ALA cows: a basal diet and supplemented prepartum with 700 g/d/cow of extruded flaxseed supplement rich in ALA C18:3n-3 (Valomega 160, Valorex, France), and PP supplemented at 6.4% of diet (dry matter basis).	60 days	Plasma and adipose tissue	↓Omega-6/Omega-3 ratio in plasma, reduced feed intake;<br>↓IL-6 and IL-17α,white blood cells;<br>↑Systemic insulin sensitivity;<br>↓Inflammatory proteins in adipose tissue:TNFα, FAAH, MGLL, RELA, AMPK.	[11]
Proteomics	C57BL/6 mice	High Omega-3 PUFAs diet: 10% Omega-3 PUFAs;<br>Low Omega-3 PUFAs diet: 2% Omega-3 PUFAs.	4 months	Blood and liver	↓TG;<br>↓Regucalcin, adenosine kinase and aldehyde dehydrogenase;<br>↑Apolipoprotein A-I, S-adenosylmethionine synthase, fructose-1, 6-bisphosphatase, ketohexokinase, malate dehydrogenase, GTP-specific succinyl CoA synthase, ornithine aminotransferase and protein disulfide isomerase-A3.	[12]
Proteomics, Lipidomics	SD rats	Control diet;<br>high-fat high-sucrose (HFHS) diet;<br>HFHS+Omega-3 PUFA (±0.8 mL/Kg body weight EPA/DHA 1:1 per week.	24 weeks	Blood and liver	↑SOD, CAT, GPx and GR and more GSH;<br>↓ARA;<br>↑EPA and DHA;<br>↓Over 20 protein carbonylation: lipid metabolism (albumin), carbohydrate metabolism (pyruvate	[13]

					carboxylase),urea cycle (carbamoyl-phosphate synthase), cytoskeleton dynamics (actin), or response to oxidative stress (catalase).	
Proteomics	Wistar Kyoto rats	Standard diets control: STD-C;<br>STD-FOM: fish oil mixture;<br>STD-GSE: grape seed polyphenols extract;<br>STD-FOM&GSE;<br>HFHS-C: high-fat and high-sucrose;<br>HFHS-FOM: fish oil mixture;<br>HFHS-GSE: grape seed polyphenols extract;<br>HFHS-FOM&GSE.	24 weeks	Plasma and liver	↓Total cholesterol level and the amount of free fatty acids (FFA), insulin concentration;<br>↑Glutathione peroxidase (GPX);<br>↓TNFα, CRP;<br>↓Carbonylation：Albumin (Alb), mitochondrial aldehyde dehydrogenase (Aldh2), mitochondrial long-chain specific Acyl-CoA dehydrogenase (Acadl), 4-hydroxyphenylpyruvate dioxygenase (Hpd) and beta-ureidopropionase (Upb1), and mitochondrial ornithine carbamoyltransferase (Otc).	[14]
Lipidomics	C57BL/6J mice	Control diet;<br>Control diet + 1% EPA-E (w/w) (EPA-fed group);<br>Control diet +1% DHA-E (w/w) (DHA-fed group);<br>Control diet +1% ARA-E (w/w) (ARA-fed group).	2 weeks	Liver, kidney, white adipose, skeletal muscle, heart, small intestine, lung, brain, and spleen, and plasma	↓Cholesterol, decreased triacylglycerol,C16:0, C18:1, C18:2;<br>↑C20:4 (ARA), C20:5 (EPA), and C22:6 (DHA),oxylipins intake of PUFAs strongly impacted the lipid profiles of metabolic organs such as the liver and kidney, while causing less impact on the brain.<br>Mouse tissues, except for the brain, effectively incorporated the dietary PUFAs into glycerolipids and glycerophospholipids, and tissue levels of free fatty acids and oxylipins were well correlated with dietary PUFA intakes	[15]
Lipidomics	C57BL/6 mice	Control diet;<br>Control diet + “Pecorino” cheese (CHE);<br>Control diet + ALA and CLA-enriched “Pecorino” cheese (ENR).	8 weeks	Liver, muscle, adipose tissue and brain	↓Saturated fat;<br>↑CLA and ALA;<br>↓Inflammatory genes.	[16]
Lipidomics	C57BL/6J mice	Chow diet;<br>HFD;<br>Flaxseed oil diet.	3 days	Serum, mesenteric adipose tissue (MAT), and liver	↓Food intake and fasting glucose;<br>↑EPA, DHA incorporation in MAT and the liver.	[17]
Transcriptomics, Lipidomics	C57BL/6J mice	F1 Omega-3 lineage:fish oil,75% of Omega-3 PUFAs;<br>Control lineage:sunflower oil,83.5% of oleic acid;<br>F3 Omega-3 lineage+HFD(Hfepa);<br>F3 Control lineage+HFD (Hfoleic);<br>F3 Control lineage+LFD (reference).	17 weeks	Gastrocnemius muscle and plasma	↓Weight, fat mass,Fasting plasma glucose and insulin,HOMA-IR index,Glucose tolerance,Insulin tolerance;<br>↑Energy expenditure,plasma glycerol and non-esterified fatty acids (NEFA);<br>↑PC 40:8 and PI 38:6;<br>↑FOXO1, FOXO3, and Nfe2l2.	[18]
Lipidomics	C57BL/6J mice	Chow diet;<br>Conventional butter (Bu,Omega-6/Omega-3=6);<br>Omega-3 enriched butter (n3Bu, Omega-6/Omega-3=1);<br>Margarine (Ma,n-6/n-3=6).	10 weeks	Blood, liver, adipose tissue, brain	↓Fat mass, fasting blood glucose and insulin levels;<br>↓TG,adipose tissue inflammation:NF-κB activation and M1 macrophage polarization;<br>↑Insulin sensitivity;<br>↑EPA and ALA, EPA-derived oxylipins.	[19]
Lipidomics	C57BL/6J mice	Low fat diet (Control);<br>HFD;<br>HFD+4% salmon phospholipids(SA4 group).	14 weeks	Serums and colonic contents	↓Fasting blood glucose (FBG), insulin, MCP-1, IL-6 and TNF-α;<br>↑Adiponectin;<br>↑TG(18:0/18:1/18: 2), TG(14:0/18:1/20:3), TG(14:0/18:0/20:2), TG(14:0/16:0/18:2),↓sphingomyelin (SM) (d20:0/24:1), SM(d18:0/ 22:0), SM(d18:0/14:0) and 5-iso PGF2VI.	[20]

Lipidomics	Male Swiss and LDLr-KO mice	Standard rodent chow (CT);<br>HFD;<br>HFD+flax seed oil (FS).	8 weeks	Blood and aorta	↓LDLc;<br>↑HDLc,ω3 (C18:3) content;<br>↓Inflammatory:IL-1β, TNF-α,pIκBα, pIKKβ;unfolded protein response markers: ATF6 and GRP78.	[21]
Lipidomics	C57BL/6 mice	Control diet;<br>HFD;<br>Omega-3 PUFA-enriched HFD.	4 days	Plasma and liver tissue	↓TG content in liver;<br>↓ACC, FAS;<br>↑Acyl-CoA oxidase and short-chain and medium-chain fatty acyl-CoA dehydrogenase: SCAD, MCAD, AOX1;<br>↓C18:2, C18:3 and C22: 4 content,ω-6 PUFA C20:4 (ARA);<br>↑Omega-3 PUFAs C20:5 (EPA) and C22:5 (DPA) content;<br>↓CCL2,IL-6 and TNFα;<br>↑HEPEs and EEQs: 17,18-EEQ, 5-HEPE and 9-HEPE.	[22]
LipidomIics, Proteomics	Wistar rats	Control:Plasmalyte *i.v.* granules;<br>ENIL:Plasmalyte i.v. Intralipid *per os*, granules;<br>ENILOV:Plasmalyte i.v. Intralipid + Omegaven *per os*, granules;<br>PNIL:nutrition mixture with Intralipid *i.v.*;<br>PNILOV:nutrition mixture with Intralipid + Omegaven *i.v.*	12 days	Liver tissue	↑EPA and DHA-containing lipid species;<br>↑CYP4A isoenzymes capable of bioactive lipid synthesis and the increased content of their potential products (oxidized EPA and DHA);<br>↓Enzymes involved CYP450 drug metabolism.	[23]
Lipidomics	C57BL/6J	HFD;<br>HFD+fish oil;<br>HFD+krill oil;	6 weeks	Liver and brain	↑TAG content;<br>↓PL;<br>↑Polyunsaturated Omega-3 fatty acids EPA (20:5) and DHA (22:6) : PC, TAG, PE and CE;DHA: CE, PC, PC O, PS and TAG;<br>↓18:2 and 20:4.	[24]
Lipidomics	C57BL/6 mice	High fat, 77% SFA;<br>Low fat, 77% SFA;<br>High fat, 77% Omega-6 PUFAs;<br>Low fat, 77% Omega-6 PUFAs;<br>High fat, 77% Omega-3 PUFAs;<br>Low fat, 77% Omega-3 PUFAs;	16 weeks	Hypothalamus, subcutaneous white adipose tissue (scWAT) and intrascapular brown adipose tissue (iBAT)	↓Weight gain, cell size;<br>↑Beige/brite adipocytes, lipotoxicity of white adipose tissue, as evidenced by increased fibrosis, lipofuscin;<br>↑Peroxidized Omega-3 PUFA diet: Peroxidation,nonenzymatic oxidised lipid metabolite;<br>↓Peroxidized Omega-3 PUFA diet: antiinflammatory cytokines il4, il10 and il13, Schwann cell marker sox10, brain derived neurotrophic factor (bdnf), expression of synaptic markers (synapsin I & II, synaptophysin, psd95,thermogenesis markers genes:ucp1, elovl3.	[25]
Lipidomics, Transcriptomics	C57BL/6N mice	cHF supplemented with phosphatidylcholine-rich concentrate from herring (replacing 10% ofdietary lipids; PC);<br>cHF containing rosiglitazone (10 mg/kg diet; R);<br>PC + R.	7 weeks	Plasma, liver and feces	↓Weight gain, TG, NEFA, TC, Fasting blood glucose, glucose tolerance, fasting plasma insulin, HOMA-IR;<br>↓Arachidonic acid (20:4n-6);<br>↑ EPA and DHA;<br>↓Hepatic biosynthetic pathways;<br>↓Lipogenic enzymes:Acly, Fasn, Scd1 and Elovl5,lipogenesis: Acacb; cholesterol biosynthesis genes: Hmgcs1, Fdps and Sqle;<br>↑FA oxidation enzymes: Ehhadh and Acox1, Acadm, Acadl and Acot1,cholesterol excretion: Scarb1 and Abcg5.	[26]
Lipidomics, MicrobiomIics, Endocannabinoid omics	Fat-1 mice, C57BL/6J mice	WT-LFD+Omega-6 PUFA:safflower oil, 2.5 μL/g/day;<br>Fat-1-LFD+Omega-6 PUFA:safflower oil, 2.5 μL/g/day;<br>WT-HFD+Omega-6 PUFA:safflower oil, 2.5 μL/g/day;<br>Fat-1-HFD+Omega-6 PUFA:safflower oil, 2.5 μL/g/day;<br>WT-HFD+Omega-3 PUFA: Fish oil, 2.5 μL/g/day;	12 weeks	Plasma, liver, and feces	↓*Lachnospiraceae g, Ruminococcaceaeg, Dorea, Proteus,* and *Coprococcus genera,*↑ *Turicibacter* and *Allobaculum* genera;<br>↑Propionate, isovalerate, isobutyrate;<br>↑17-HDHA, 18-HEPE;<br>↑2-EPG, EPEA, E-DPG, 2-DHG, DHEA;<br>↓2-LG, LEA, 2-AG, AEA.	[27]

Lipidomics	C57BL/6 mice	Chow diet;<br>Chow diet+ 5% w/w of EPA ethyl ester.	2 weeks	Bone marrow	↑17,18-EpETE;12-OH-17,18-EpETE;<br>↓LTB4-induced PMN chemotaxis and polarization,PMN infiltration.	[28]
Metabolomics, Lipidomics	C57BL/6 mice	Chow diet;<br>Chow diet+DL-Hcy;<br>Chow diet+DL-Hcy+Omega-3 PUFAs (DHA:EPA= 3:1).	6 weeks	Liver and plasma	↓Lipid accumulation, liver weight, liver/body mass ratios, hepatic TG and TC;<br>↓Fasn, Srebp1c and Pparγ;<br>↓Ceramides;<br>↓Sptlc3 and Degs2.	[29]
Metabolomics	C57BL/6J mice	LFD (10% of energy from fat);<br>HFD (45% of energy from fat);<br>HFEPA-P: HFD+EPA (45% of energy from fat; 36 g/kg EPA; HFD+EPA),11 weeks;<br>HFD-EPA-R: HFD for 6 weeks + HFD+EPA diet for 5 weeks	11 weeks	Adipose tissue	↓bodyweight, adiposity, adipocyte size, and macrophage infiltration, inflammation (MCP-1 expression);<br>↑Omega-3 PUFAs, such as EPA, Acetylcarnitine;<br>↓Omega-6 PUFAs (Figure 3C) and their PG metabolites, spermidine;<br>↓Aipogenesis/lipogenesis (e.g., Bmp4 and Cebpa); lipid accumulation (e.g., Lep and Srebf1).	[30]
Metabolomics	SD rats	Control (K) group:Common Diet;<br>Model (M) group:HFD;<br>Low-dose (GA) group: HFD and low dose LYCRPLs (0.005 g/mL);<br>Medium-dose group (GB): HFD and medium-dose LYCRPLs (0.015 g/mL);<br>High-dose (GC) group: HFD and high-dose LYCRPLs (0.03 g/m);<br>Large yellow croaker roe phospholipids(LYCRPLs).	every day for 8 weeks	Feces	GA vs. M:↑glycolic acid, L-cysteine, and glycerol 3-phosphate,↓3-methyl-2-oxovaleric acid and phosphoenolpyruvic acid;<br>GB vs. M:↑Lcysteine, L-glutamine, and pantothenic acid;<br>GC vs. M:↑L-cysteine, D-glucose, pantothenic acid, L-lysine, and glycerol 3-phosphate,↓3-methyl-2-oxovaleric acid and oxoglutaric acid;<br>Pathway: pyrimidine metabolism, the TCA cycle, the metabolism of L-cysteine, carnitine synthesis, pantothenate and CoA biosynthesis, glycolysis, and bile secretion.	[31]
Metabolomics	C57BL/6 mice	Normal diet:10% kcal fat;<br>HFD: SFA, lard, 60% kcal fat;<br>Low Omega-3/Omega-6 ratio diet: safflower oil, 60% kcal fat, safflower oil:lard=50%:50%;<br>Middle Omega-3/Omega-6 ratio diet: fish oil, 60% kcal fat, fish oil:safflower oil:lard=25%:25%:50%;<br>High Omega-3/Omega-6 ratio diet: fish oil, 60% kcal fat, fish oil:lard=50%:50%.	12 weeks	Blood and liver	↓Weight,glucose level, TC, LDL-C, atherosclerosis index;<br>↑Free fatty acid;<br>↓Fumaric acid, oxidative stress;<br>↓mTORC1 pathway,fatty acid synthesis(FAS, PPARα, PPARγ and SREBP-1c) and oxidation(ACC1 and ACC2);<br>↑Mitochondrial electron transport chain and TCA.	[32]
Metabolomics	Wistar rats	Control offspring:7% soybean oil;<br>GDM offspring:7% soybean oil;<br>Omega-3 PUFA adequate GDM(Gestational diabetes mellitus) offspring:3% soybean oil+4%fish oil;<br>Omega-3 PUFA deficient GDM offspring:7% safflower oil.	10 months	Liver and blood	↓Triglyceride and total cholesterol,oxidative stress and inflammation(IL-1β and increased IL-10), TC/HDL, GTT , ITT;<br>↑SOD and CAT;<br>↓α-linolenic acid, 9′-Carboxy-γ -tocotrienol, Oxalacetic acid, Phenylethylamine.	[33]
Metabolomics	C57BL/6 or Fat-1 transgenic mice	Sham;<br>MI (myocardial infarction);<br>Sham+FO;<br>FAT-1 sham;<br>FAT-1 MI.	3 weeks	Plasma	↑19,20-EDP and 17,18-EEQ;<br>↓9,10-EpOME;<br>Omega-3 PUFA-rich diets feeding or transgene of Fat-1 shifted the eicosanoid profile to an Omega-3 PUFA dominant style and attenuated the myocardial infarction injury.	[34]
Microbiomics	C57BL/6 mice	Chow diet (Chow);<br>Chow+beef diet (Beef);	60 days	Serum and feces	↓serum triglyceride levels;<br>↓*Escherichia-Shigella, Mucispirillum, Helicobacter,* and	[35]

		Chow+Omega-3 diet (Cw3);<br>Chow+beef+Omega-3 diet (Bw3).			*Desulfovibrio;*<br>↑Energy and glucose metabolism.	
Microbiomics	Wistar rats	Control diet fecal sample+Fish oil (omega-3 source):2%(w/v);<br>Control diet fecal sample+Pomegranate oil (punicic acid source):2%(w/v);<br>Control diet fecal sample+a mixture of both oils :2%(w/v);<br>High-fat high-sugar diet (WD) fecal sample+Fish oil (omega-3 source):2%(w/v);<br>High-fat high-sugar diet fecal sample+Pomegranate oil (punicic acid source):2%(w/v);<br>High-fat high-sugar diet fecal sample+a mixture of both oils:2%(w/v);<br>FOS (Positive control);<br>High-fat high-sugar diet fecal sample (Negative control)<br>Control diet fecal sample (Negative control).	0,6,12,24 and 48h	Feces	↑α-diversity, *Firmicutes, Bacteroidetes, Akkermansia, Blautia;*<br>↑GABA, tyrosine concentration.	[36]
Microbiomics	BALB/c mice	Lard obesity-inducing diet (Lard OID);<br>Lard+flaxseed oil diet (Flaxseed OID);<br>High sugar diet (HS);<br>Control diet.	4 weeks	VAT	↓body and VAT weight, adipocyte diameter and fibrosis;<br>↑VAT hippurate;<br>↑*Rikenellaceae, Clostridium,* and *Oscillospira.*	[37]
Microbiomics	C57BL/6 mice	DHEA-induced PCOS mouse model+Omega-3 PUFAs(2 g/kg every 2 days);<br>DHEA-induced PCOS mouse model+corn oil (2 g/kg every 2 days).	8 weeks	Feces, subcutaneous adipose tissues, serum	↓Alloprevotella;<br>↑Akkermansia, Alistipes;<br>↓IL-1β, TNF-α and IL-18 (inflammatorty cytokines);<br>↓Ucp1, Pgc1a, Cited and Cox8b (thermogenic markers);<br>↓Fasting blood glucose levels, GTTs and ITTs.	[38]
Microbiomics	SD rats	Control (K) group: Common Diet;<br>Model (M) group: HFD;<br>Low-dose (GA) group: HFD and low dose LYCRPLs (0.005 g/mL);<br>Medium-dose group (GB): HFD and medium-dose LYCRPLs (0.015 g/mL);<br>High-dose (GC) group: HFD and high-dose LYCRPLs (0.03 g/mL);<br>Positive control group (Y): Basic feed and simvastatin (0.02 mg/mL);<br>Large yellow croaker roe phospholipids (LYCRPLs).	0, 2, 4, 6, and 8 weeks	Blood, intestinal contents, liver, perirenal fat, and epididymal fat	↓Weight, perirenal fat index, epididymal fat index, TG, TC, and LDL-C;<br>↑HDL-C, size and number of hepatocytes, number of adipocytes in epididymal fat;<br>↑Biosynthesis of 12-, 14- and 16-membered macrolides pathways, Glycosphingolipid biosynthesis lacto and neolacto series, as well as steroid hormone biosynthesis;<br>↓*Prevotella* (OTU1131, OTU420 and OTU3734), *Mucispirillum* (OTU248) and *Alistipes* (OTU1682, OTU1207 and OTU1042);<br>↑*Bacteroides* (OTU602 and OTU236) and *Alloprevotella* (OTU772);<br>↓*Proteobacteria, Actinobacte* and *Desulfovibrionaceae.*	[39]
Microbiomics, Lipidomics	SD rats	Pair-fed (PF) with corn oil (CO) group (PF/CO):pair-fed (PF) group were i.p injected with an equal volume of citrate buffer and normal saline;<br>DM(T2DM) with CO group (DM/CO):corn oil (CO);<br>PF with FO group (PF/FO):flaxseed oil (FO);<br>DM with FO group (DM/FO).	5 weeks	Serum and feces	↑*Bacteroidetes* and *Alistipes*;<br>↓*Firmicutes* and *Blautia*, the ratio of *Bacteroidetes-Firmicutes*;<br>↓Fasting blood glucose (FBG) and Glycated hemoglobin (GHb), TC, TG and LDL, MDA, LPS levels;<br>↑HDL, SOD levels;<br>↓IL-1β, TNF-α, IL-6 and IL-17A;<br>↑Acetic acid, propionic acid and butyric acid.	[40]

Microbiomics	C57BL/6J mice	Control+high Omega-6/Omega-3 ratio diet (LA/ALA ratio:15);<br>ELS (Early-Life Stress) conditions+low Omega-6/Omega-3 ratio diet (LA/ALA ratio:1.1);<br>Control+low Omega-6/Omega-3 ratio diet (LA/ALA ratio:1.1)<br>ELS conditions+high Omega-6/Omega-3 ratio diet (LA/ALA ratio:15).	40 days	Feces	↓*Firmicutes Erysipelotrichia:Erysipelotrichia, Erysipelotrichales*, and *Erysipelotrichaceae;*<br>At P42, species abundance correlated with body fat mass and circulating leptin (e.g., *Bacteroidetes* and *Proteobacteria* taxa) and fatty acid profiles (e.g., *Firmicutes* taxa).	[41]
Lipidomics, Microbiomics	C57BL/6J mice	Control lean diet;<br>HFD;<br>HFD+EPA.	6, 13 weeks	Feces, white adipose tissue, blood	↓Fat mass, fasting glucose, fasting insulin;<br>↑EPA-derived metabolites: 5-, 8-, 9-, 11- and 15-HEPE; DHA-derived metabolites: 14-HDHA, 17-HDHA, 19,20-DiHDPA and 11-HDoHE; lipid mediators: 17(18)-EpETE and 17,18-DiHETE;<br>↓IL-6 and leptin levels;<br>13 weeks:↓*Lactobacillus*, ↑*Verrucomicrobia* phylum, *Alcaligenacae and Sutterella* family, as well as the *Akkermansia* genus;<br>6 weeks:↑*Bacteroidetes*, *Proteobacteria* and *Verrucomicrobia* , *Bacteroidia, Betaproteobacteria, Bacteroidales, Alcaligenaceae* , *Sutterella* and *Akkermansia*;↓*Lactobacillales, Lachnospiraceae, Ruminococcaceae.*	[42]
Microbiomics	C57BL/6J mice	Cohort 1: CTRL; HFD-sat (saturated fat); HFD-Omega-6; HFD-Omega-3;<br>Cohort 2: CTRL-veh(saline); CTRL-RvD1-H(high-dose resolvin D1);HFD-sat-veh; HFD-sat-RvD1-L(low-dose resolvin D1); HFD-sat-RvD1-H; HFD-sat-n3.	8 weeks	Feces, serum, adipose tissue, colon	↓Weight, fat, HOMA-IR, inflammation;<br>↑Transepithelial resistance;<br>↓H2S-producing bacteria: *Bilophila* and *Desulfovibrio.*	[43]
Microbiomics	C57BL/6 mice	HFD:41.2% kcal lard+18.7% kcal corn oil;<br>HFD+FO:41.2% kcal lard+13.4% kcal corn oil+5.3% kcal fish oil.	12 weeks	Feces, blood, spleen, cecum, adipose tissue, ileum, colon	↑LC fatty acids EPA (20:5n-3) and DHA (22:6n-3);<br>↓Linoleic acid (18:2n-6) and arachidonic acid (20:4n-6), HOMA-IR, serum glucose concentrations;<br>↓Inflammatory hormones leptin and resistin, visceral adipose tissue depot weights;<br>↑Insulin sensitizing and anti-inflammatory adipokine, adiponectin;<br>↓*Firmicutes* phylum: *Ruminococcaceae* and *Lachnospiraceae* families, *Dehalobacterium* genus, and *Ruminococcus* gnavus<br>↑*Firmicutes* phylum: *Christensenellaceae* genera, *Erysipelotrichaceae, Peptostreptococcaceae* and *Clostridiaceae* families and the *Allobaculum, Dorea* and *Ruminococcus* genera; *Bacteroidetes* phylum: *Bacteroidales* order and *Rikenellaceae* family; *Akkermansia muciniphila*; *Bacteroidetes, Proteobacteria*, TM7, *Verrucomicrobia, Cyanobacteria* and *Tenericutes*.	[44]

Table S2. Omics research on human subjects.

Omic Type	Sample	Group/Intervention	Duration	Tissue Evaluated	Main outcome	Ref.
Genomics	191 Greenlandic Inuit, 60 individuals of European ancestry (CEU) and 44 Han Chinese individuals (CHB).	Greenlandic Inuit;<br>European ancestry individuals (CEU);<br>Han Chinese individuals (CHB).	/	DNA sample	Allele-frequency difference was found in a cluster of fatty acid desaturases-FADS1,FADS2,andFADS3.	[45]
Epigenomics	36 overweight and obese subjects.	3 g of Omega-3 PUFAs (including 1.9–2.2 g of EPA and 1.1 g of DHA).	6 weeks	Blood	↓TG, TC,TC/HDL-C, Omega-6 FAS;<br>↑Omega-3 FAS, EPA, DHA, Omega-3/Omega-6 ratio;<br>308 CpG sites, assigned to 231 genes, were differentially methylated;<br>16/55 pathways were related to inflammatory and immune response, lipid metabolism, T2DM, and cardiovascular signaling;<br>CpG sites within AKT3, ATF1, HDAC4, and IGFBP5 were correlated with plasma TG, glucose levels and total cholesterol/HDL-cholesterol ratio.	[46]
Epigenomics	118 healthy mother–newborn pairs.	Low Omega-3 PUFA (n=144, n-3 PUFA concentration <25th percentile);<br>Medium (n=269, Omega-3 PUFAs between 25th and 75th percentiles);<br>High Omega-3 PUFA content (N=144, Omega-3-3 PUFAs >75th percentile).	9-10 months	Blood	Differentially methylated genes: MSTN, IFNA13, ATP8B3, and GABBR2, that are involved in the onset of insulin resistance and adiposity, innate immune response, phospholipid translocation across cell membranes, and mechanisms of addiction to high fat diet, alcohol, and sweet taste.	[47]
Transcriptomics	Human umbilical vein endothelial cells (HUVECs).	HUVECs+DHA, 0 h; IL-1β, 0h;<br>HUVECs+50 μmol/L DHA 48h;<br>HUVECs+IL-1β, 3h;<br>HUVECs+50 μmol/L DHA 48h +IL-1β,3h.	48 h	HUVECs	↓Transforming growth factor (TGF)-β2, angiopoietin (ANGPT)-1 and CD47;<br>↑Cytochrome P 450 (CYP) 4F2.	[48]
Transcriptomics	8 participants.	Participants+olive;<br>Participants+nuts;<br>Participants+Omega-3 (Fish oil or EPA+DHA).	/	Peripheral blood mononuclear cells (PBMC)	↓NFIL3, IL8, STK17B, SERPINB2, and RGS;<br>↓HNF4A, IRF1, REST, CTCF, and SREBF2;<br>↑miR-17-5p, miR-335-5p, miR-93-5p.	[49]
Transcriptomics	76 women with obesity	Patients+Omega-3 PUFAs (1.8 g/day EPA+DHA);<br>patients+placebo capsules.	3 months	Blood	↓Weight, adipose tissue mass and BMI;<br>↑EPA and DHA in plasma phosphatidylcholine,pro-resolving DHA derivatives;<br>↓Inflammatory markers (SELE, MCP-1, sVCAM-1, sPECAM-1, and hsCRP), fasting triglycerides and insulin;<br>↑PPAR-α (SLC25A20, HADHB, HADH, GCDH, ACAT1, DECR1), NRF2 and NF-κB (NFKBIA, NFKBIB, NFKBIZ) target genes.	[50]

Transcriptomics	Normal weight, n=3<br>Obese group, n=3<br>CRC group, n=3	Normal weight group + DHA (10 μM);<br>Normal weight group + AA (5 μM);<br>Obese group + DHA (10 μM);<br>Obese group + AA (5 μM);<br>CRC group + DHA (10 μM);<br>CRC group + AA (5 μM).	18 h	VAT	↓lncRNA: LUCAT1, PSMG3-AS1;<br>↑Calnexin/calreticulin cycle(DHA);<br>↑DHA: Immunity and infection (e.g., antigen processing, IL-1 signaling), cancer-related pathways (e.g., TP53 activity, PTEN regulation, TGFβ signaling), extracellular matrix organization and fibrosis (e.g., collagen formation);<br>↑AA: oncogenic MAPK pathway, EGFR signaling (e.g., signaling by EGFR, EGFR down-regulation), metabolism (e.g., Triglyceride metabolism, the synthesis of PC), inflammation and oxidative stress (e.g., TNF and Interleukin-17 signaling, the metabolism of nitric oxide), and signaling pathways that play pivotal roles in the oncogenic process (e.g., FGFR1-4 signaling, NOD1/2 signaling).	[51]
Proteomics	75 Metabolic syndrome(MetS) patients	High-SFA (HSFA, high saturated fatty acids) diet: n=17;<br>High-MUFA (HMUFA) diet: n=18;<br>Low-fat, high complex carbohydrate (LFHCC) diet + oleic sunflower oil: n=20;<br>Low-fat, high complex carbohydrate diets +long-chain Omega-3 PUFA (LFHCC n-3): n=20.	12 weeks	Adipose tissue	↓Insulin resistance, HOMA-IR,NEFA levels, insulin levels;<br>↓mRNA: EHD2, GAPDH;<br>↑ mRNA: CAP;<br>↓Protein: Gelsolin isoform 32, GPD1 (soluble), Anxa2.	[52]
Proteomics, Lipidomics	24 MetS patients	High-saturated fatty acid (HSFA);<br>High-monounsaturated fatty acid (HMUFA);<br>Low-fat, high-complex carbohydrate diets supplemented with oleic sunflower oil (LFHCC);<br>Low-fat, high-complex carbohydrate diets supplemented with long chain (LC); Omega-3 PUFAs (LFHCC n-3).	12 weeks	Blood	↑VPS28, BiP, S4-SRCRB, GSN;<br>↓CLIC1, ACTB, POTE-2, CAPZ, HSP70-2, MACF1.	[53]
Proteomics	103 patients with non-alcoholic steatosis or steatohepatitis	Participants + 3.36 g daily of DHA &EPA (1 g of Omacor contains 460 mg of EPA and 380 mg of DHA as ethyl esters);<br>Participants + 4 g of olive oil (placbo group)	15-18 months	Blood	↓Prothrombin and apolipoprotein B-100.	[54]
Proteomics	41 overweight aldults (aged 23 to 44	EPA-Rich Fish Oil Supplement (EPA/DHA: 2.3, EPA: 3.5 g; DHA: 1.5 g)<br>DHA-Rich Fish Oil Supplement (EPA/DHA: 0.3, EPA: 1 g; DHA: 3.6 g).	8 weeks	Plasma	↓TG, TG-related NMR parameters (VLDL-Z, VLDL-P, and TRL-P);<br>↓HDL proteins related to inflammation;<br>↑apoM abundance.	[55]

Proteomics, Lipidomics	60 NASH patients	Participants+ Omega-3 PUFAs capsules (0.945 g, 64% ALA, 21% EPA, and 16% DHA);<br>Participants + placebo capsules (mineral oil).	6 months	Liver and plasma	↑ER stress: HSPD1, EEF1A2, HNRPU, EEF2, RS27A, RL40, UBB;<br>↑Lipid metabolism: PGRMC2, FABPL;<br>↑Cellular respiration pathways: PPIA, TPI1, ALDOB, GAPDH, PGM1, ENO3, KPYR, PCKGM, LDHC, C1TC, ATP1A4, ATP1A1, CATA, ATPA, CLUD1, CLUD2, FASTKD2, CP2A6 ;<br>↑Cell matrix: FIBB, K1C9, PDIA6, TBA3E, K2C75, LMNA, K2C6B;<br>↑Plasma concentrations of ALA, EPA and DHA;<br>↓Plasma concentrations of AA.	[56]
Proteomics	32 healthy participants with overweight or grade 1 obesity (BMI 25–35 kg/m2)	Omega3-supplemented milk (131.25 mg EPA + 243.75 mg DHA/250 mL of milk)<br>PhyS-supplemented milk (1.6 g of plant sterols/250 mL of milk)	28 days	Blood	↑apolipoprotein (Apo)-E LDL content;<br>↑HDL-associated proteins: Apo A–I, lecitin cholesterol acyltransferase (LCAT), paraoxonase-1 (PON-1), Apo D, and Apo L1.	[57]
Lipidomics, Proteomics	40 participants aged between 40 and 70 y (median 64 y) with moderate Cardiovascular disease(CVD) risk, comprising 24 males and 16 females	Patients+fish oil (1.9 g/day Omega-3 PUFAs:1080 mg EPA and 810 mg DHA);<br>Patients+control oil (high-oleic safflower oil:740 mg oleic acid plus 120 mg linoleic acid).	12 weeks	Blood	↓Plasma TG concentration, systolic blood pressure(SBP);<br>↑LDL-C; Omega-3 PUFAs in plasma phosphatidylcholine and phosphatidylethanolamine;<br>↓Numbers of circulating EVs, thrombin generation, clot growth, clot size;<br>↓Proinflammatory and/or proatherosclerotic proteins:RBP4, PF4V1, PF4V1, PF4V1.	[58]
Metabolomics	20 overweight and obese patients	Patients + 3.7 g/day-1 n-3 fatty acids (1.7 g day-1 EPA and 1.2 g/day DHA);<br>Patients + 200 mg fenofibrate;<br>Patients + placebo (High Oleic Sunflower Oil).	6 weeks	Blood	↓Saturated TG-species;<br>↑Unsaturated TG-, LPC-, phosphatidylcholine-, and cholesterol ester-species.	[59]
Metabolomics	96 adult participants with Non-alcoholic fatty liver disease (NAFLD, aged 30-67 years)	PSE treatment alone group (PSE, 3.3 g of PSE, equivalent to 2.5 g phytosterol in the free form;<br>Omega-3 PUFA treatment alone group (FO, 450 mg of EPA and 1500 mg of DHA);<br>Combination of PSE and Omega-3 PUFA treatment group (FO + PSE);<br>Control group (placebo).	12 weeks	Blood	↑PUFA-containing phosphatidylcholine (PC), lysophosphatidylcholine (LysoPC), perillyl alcohol and retinyl ester.	[60]
Metabolomics	21 subjects (9 men and 12 postmenopausal women) with chronic inflammation and some characteristics of MetS.	Participants + EPA (3 g/day);<br>Participants + DHA (3 g/day);<br>Participants + high oleic acid sunflower oil (3 g/day).	10 weeks	Blood	↑EPA: serum concentrations of lactate dehydrogenase;<br>↑DHA: serum albumin concentrations;<br>↓EPA: TCA cycle intermediates fumarate and α-ketoglutarate;<br>↑EPA: glucuronate, UDP-glucuronate, and non-esterified DHA;<br>↓DHA: TCA cycle intermediates pyruvate, citrate, isocitrate, fumarate, α-ketoglutarate, and malate;<br>↑ DHA: succinate and glucuronate.	[61]

Metabolomics	12 young adults (18–35 years) and 12 older adults (65–85 years)	Young adults+Omega3-PUFAs (3.9 g/ day, 2.7 g EPA, 1.2 g DHA)<br>Older adults+Omega-3 PUFAs (3.9 g/ day, 2.7 g EPA, 1.2 g DHA)	4 months	Blood	↓TG, VLDL particle number;<br>↑HDL;<br>↑Hydroxyproline, 3-carboxy-4-methyl-5-propyl-2-furanpropionic acid (CMPF);<br>↓Kynurenine.	[62]
Metabolomics	40 Hispanic adults diagnosed with Type 2 Diabetes Mellitus(T2DM) aged 33-74 years	Neuropathic pain symptoms+2,000 mg Omega-3 fish-oil (1000 mg DHA and 200 mg EPA);<br>No neuropathic pain symptoms+2,000 mg Omega-3 fish-oil (1000 mg DHA and 200 mg EPA).	12 weeks	Blood	↑EPA, DHA;<br>↓Sphingosine levels.	[63]
Lipidomics	Overweight subjects:20 subjects (8 men, 12 women) with an average age of 50.6 ± 1.8 years	Patients+Omega-3 supplemented milk (131.25 mg EPA + 243.75 mg DHA/250 mL of milk; total Omega-3 supplement 375 mg/day);<br>Patients+PhyS-supplemented milk (1.6 g of plant sterols/250 mL of milk; total supplement 1.6 g/day).	12 weeks	Blood	↓Lipids esterified with arachidonic acid (AA) (PC 17:0/20:4, PC 20:3/20:4, PC O-18:0/20:4, PE 16:0/20:4, PE 18:0/20:4 and PI 16:0/20:4);<br>↑Lipids containing DHA (e.g., PC-DHA, PC 15:0/22:6, PC 16:0/22:6, PC 18:0/22:6, TAG 58:8 and CE 22:6);lipids esterified with EPA e.g., PC (related to lipid metabolism).	[64]
Lipidomics	60 patients with MetS and NAFLD	Patients + Omega-3 PUFAs (EPA 215 mg+DHA 155 mg);<br>Patients + placebo (450 mg of soya oil).	12 months	Plasma	↓GGT activity<br>↑23 lipids were increased, and 21 of them contained one of the Omega-3 PUFAs: 3 TGs, 2 FFAs, and 16 phospholipids	[65]
Lipidomics	12 normal-weight (BMI 22.1 ± 2.3 kg/m2) healthy participants (six male). The mean age was 24 ± 2 years	Before supplementation with fish-oil capsules (Nutrifynn caps, 2 g/day, containing 200–250 mg EPA and 150–200 mg DHA for each capsule);<br>After supplementation with fish-oil capsules.	0, 3, 7, 14, 21 days	Blood	↑Lysophospholipids(day 3), phosphatidylserines (later stage);<br>↓Phosphatidylcholines and alkylphosphatidylcholines (day 21).	[66]
Lipidomics	21 MetS patients	Caloric restriction group (CR);<br>CR + fish oil (CRF).	12 weeks	Blood	↓TG concentration, body weight, waist circumference, Blood pressure, Inflammatory(CRP, IL-6);<br>↑long-chain polyunsaturated fatty acids, TG (60:9) and phosphatidylcholine (p40:6).	[67]
Lipidomics	78 subjects with a high BMI, abdominal obesity, and at least one other feature of the MetS	Participants + low in Omega-3 PUFAs and polyphenols diet (Control group);<br>Participants+rich in Omega-3 PUFAs and low in polyphenols diet(Hn3Lpo group);<br>Participants +low in Omega-3 PUFAs and rich in polyphenols diet(Ln3Hpo group);<br>Participants+ rich in both Omega-3	8 weeks	Blood	↑Long-chain PUFA-containing TGs after the high Omega-3 FA diets, TG(60:10), TG(60:11), TG(62:13);<br>↑Medium-chain PCs after the diet exclusively rich in polyphenols.	[68]

		PUFAs and polyphenols diet(Hn3Hpo group)				
Lipidomics	30 healthy sedentary participants aged 30 to 50 years	Participants + normal caloric diet enriched with PUFAs (30 g/day of almonds and walnuts)	8 weeks	Blood	↓miR-328, miR-330-3p, miR-221 and miR-125a-5p;<br>↑miR-192, miR-486-5p, miR-19b, miR-106a, miR-769-5p, miR-130b and miR-18a;<br>↑Linoleic (C18:2, ω-6), eicosapentoenoic (C20:5, ω-3) and docosahexaenoic (C22:6, ω-3) PUFAs;<br>↓SaFAs (SaFAs).	[69]
Lipidomics	69 T2DM patients	Patients +5 g/day corn oil (Placebo);<br>Patients +15 mg/day pioglitazone (Pio);<br>Patients + 5 g/day EPA + DHA concentrate (Omega-3; EPAX 1050TG, EPAX AS, containing about 15 % EPA, 40 % DHA, wt/wt; i.e., 2.8 g EPA + DHA);<br>Patients +pioglitazone + EPAX 1050TG (Pio& Omega-3).	24 weeks	Blood	↑EPA + DHA content in serum phospholipids, insulin sensitivity(Pio & Omega-3).	[70]
Microbiomics	75 participants with marginal hyperlipidemia	Patients + Omega-3 PUFAs-enriched plant oil (4 g of Omega-3 PUFAs-rich oil);<br>Patients + corn oil.	12 weeks	Blood and feces	↑DPA, EPA;<br>↓TC concentrations ;<br>↑*Bacteroidetes* phylum, *Bacteroidia* genus, *Bacteroidales* genus;<br>↓*Firmicutes* phylum, *Firmicutes* and *Bacteroidetes* ratio, *Phascolarctobacterium* genus, *Veillonella* genus.	[71]
Microbiomics	25 participants with MetS risk	Participants + canola oil (Canola; 63% MUFA, 20% LA, 10% (ALA));<br>Participants + DHA enriched canola-oil (CanolaDHA; 64% MUFA, 13% LA, 6% DHA);<br>Participants + high OA canola oil (CanolaOleic; 72% MUFA, 15% LA, 2% ALA);<br>Participants + a blend of corn oil/safflower oil (CornSaff; 18% MUFA, 69% LA);<br>Participants + a blend of flax oil/safflower oil (FlaxSaff; 18% MUFA, 38% LA, 32% ALA).	30 days	Feces	↑MUFA: *Parabacteroides, Prevotella, Prevotella, Prevotella;*<br>↑PUFA: *Isobaculum.*	[72]

References

1. Varshney, R.; Das, S.; Trahan, G.D.; Farriester, J.W.; Mullen, G.P.; Kyere-Davies, G.; Presby, D.M.; Houck, J.A.; Webb, P.G.; Dzieciatkowska, M.; et al. Neonatal intake of Omega-3 fatty acids enhances lipid oxidation in adipocyte precursors. *iScience* **2023**, *26*, 105750, doi:10.1016/j.isci.2022.105750.
2. Ballester, M.; Quintanilla, R.; Ortega, F.J.; Serrano, J.C.E.; Cassanye, A.; Rodriguez-Palmero, M.; Moreno-Munoz, J.A.; Portero-Otin, M.; Tibau, J. Dietary intake of bioactive ingredients impacts liver and adipose tissue transcriptomes in a porcine model of prepubertal early obesity. *Sci Rep* **2020**, *10*, 5375, doi:10.1038/s41598-020-62320-4.
3. Pinel, A.; Rigaudiere, J.P.; Morio, B.; Capel, F. Adipose Tissue Dysfunctions in Response to an Obesogenic Diet Are Reduced in Mice after Transgenerational Supplementation with Omega 3 Fatty Acids. *Metabolites* **2021**, *11*, 18, doi:10.3390/metabo11120838.
4. Kunz, H.E.; Dasari, S.; Lanza, I.R. EPA and DHA elicit distinct transcriptional responses to high-fat feeding in skeletal muscle and liver. *Am J Physiol Endocrinol Metab* **2019**, *317*, E460-E472, doi:10.1152/ajpendo.00083.2019.
5. Manaig, Y.J.Y.; Criado-Mesas, L.; Esteve-Codina, A.; Marmol-Sanchez, E.; Castello, A.; Sanchez, A.; Folch, J.M. Identifying miRNA-mRNA regulatory networks on extreme n-6/n-3 polyunsaturated fatty acid ratio expression profiles in porcine skeletal muscle. *PLoS One* **2023**, *18*, e0283231, doi:10.1371/journal.pone.0283231.
6. Meng, Q.; Ying, Z.; Noble, E.; Zhao, Y.; Agrawal, R.; Mikhail, A.; Zhuang, Y.; Tyagi, E.; Zhang, Q.; Lee, J.H.; et al. Systems Nutrigenomics Reveals Brain Gene Networks Linking Metabolic and Brain Disorders. *EBioMedicine* **2016**, *7*, 157-166, doi:10.1016/j.ebiom.2016.04.008.
7. Soni, N.K.; Nookaew, I.; Sandberg, A.S.; Gabrielsson, B.G. Eicosapentaenoic and docosahexaenoic acid-enriched high fat diet delays the development of fatty liver in mice. *Lipids Health Dis* **2015**, *14*, 74, doi:10.1186/s12944-015-0072-8.
8. Lopez-Dominguez, J.A.; Canovas, A.; Medrano, J.F.; Islas-Trejo, A.; Kim, K.; Taylor, S.L.; Villalba, J.M.; Lopez-Lluch, G.; Navas, P.; Ramsey, J.J. Omega-3 fatty acids partially revert the metabolic gene expression profile induced by long-term calorie restriction. *Exp Gerontol* **2016**, *77*, 29-37, doi:10.1016/j.exger.2016.02.002.
9. Soni, N.; Ross, A.B.; Scheers, N.; Nookaew, I.; Gabrielsson, B.G.; Sandberg, A.S. The Omega-3 Fatty Acids EPA and DHA, as a Part of a Murine High-Fat Diet, Reduced Lipid Accumulation in Brown and White Adipose Tissues. *Int J Mol Sci* **2019**, *20*, 18, doi:10.3390/ijms20235895.
10. Corral-Jara, K.F.; Cantini, L.; Poupin, N.; Ye, T.; Rigaudiere, J.P.; De Saint Vincent, S.; Pinel, A.; Morio, B.; Capel, F. An Integrated Analysis of miRNA and Gene Expression Changes in Response to an Obesogenic Diet to Explore the Impact of Transgenerational Supplementation with Omega 3 Fatty Acids. *Nutrients* **2020**, *12*, 20, doi:10.3390/nu12123864.
11. Kra, G.; Daddam, J.R.; Moallem, U.; Kamer, H.; Mualem, B.; Levin, Y.; Kocvarova, R.; Nemirovski, A.; Contreras, A.G.; Tam, J.; et al. Alpha-linolenic acid modulates systemic and adipose tissue-specific insulin sensitivity, inflammation, and the endocannabinoid system in dairy cows. *Sci Rep* **2023**, *13*, 5280, doi:10.1038/s41598-023-32433-7.
12. Ahmed, A.A.; Balogun, K.A.; Bykova, N.V.; Cheema, S.K. Novel regulatory roles of omega-3 fatty acids in metabolic pathways: a proteomics approach. *Nutr Metab (Lond)* **2014**, *11*, 6, doi:10.1186/1743-7075-11-6.

13. Munoz, S.; Mendez, L.; Dasilva, G.; Torres, J.L.; Ramos-Romero, S.; Romeu, M.; Nogues, M.R.; Medina, I. Targeting Hepatic Protein Carbonylation and Oxidative Stress Occurring on Diet-Induced Metabolic Diseases through the Supplementation with Fish Oils. *Mar Drugs* **2018**, *16*, 23, doi:10.3390/md16100353.
14. Mendez, L.; Munoz, S.; Miralles-Perez, B.; Nogues, M.R.; Ramos-Romero, S.; Torres, J.L.; Medina, I. Modulation of the Liver Protein Carbonylome by the Combined Effect of Marine Omega-3 PUFAs and Grape Polyphenols Supplementation in Rats Fed an Obesogenic High Fat and High Sucrose Diet. *Mar Drugs* **2019**, *18*, 30, doi:10.3390/md18010034.
15. Naoe, S.; Tsugawa, H.; Takahashi, M.; Ikeda, K.; Arita, M. Characterization of Lipid Profiles after Dietary Intake of Polyunsaturated Fatty Acids Using Integrated Untargeted and Targeted Lipidomics. *Metabolites* **2019**, *9*, 17, doi:10.3390/metabo9100241.
16. Tognocchi, M.; Conte, M.; Testai, L.; Martucci, M.; Serra, A.; Salvioli, S.; Calderone, V.; Mele, M.; Conte, G. Supplementation of Enriched Polyunsaturated Fatty Acids and CLA Cheese on High Fat Diet: Effects on Lipid Metabolism and Fat Profile. *Foods* **2022**, *11*, 21, doi:10.3390/foods11030398.
17. Nakandakari, S.; Gaspar, R.C.; Kuga, G.K.; Ramos, C.O.; Vieira, R.F.; Rios, T.D.S.; Munoz, V.R.; Sant'ana, M.R.; Simabuco, F.M.; da Silva, A.S.R.; et al. Short-term flaxseed oil, rich in omega 3, protects mice against metabolic damage caused by high-fat diet, but not inflammation. *J Nutr Biochem* **2023**, *114*, 109270, doi:10.1016/j.jnutbio.2023.109270.
18. Pinel, A.; Rigaudiere, J.P.; Jouve, C.; Montaurier, C.; Jousse, C.; M, L.H.; Morio, B.; Capel, F. Transgenerational supplementation with eicosapentaenoic acid reduced the metabolic consequences on the whole body and skeletal muscle in mice receiving an obesogenic diet. *Eur J Nutr* **2021**, *60*, 3143-3157, doi:10.1007/s00394-021-02502-6.
19. Fan, R.; Kim, J.; You, M.; Giraud, D.; Toney, A.M.; Shin, S.H.; Kim, S.Y.; Borkowski, K.; Newman, J.W.; Chung, S. alpha-Linolenic acid-enriched butter attenuated high fat diet-induced insulin resistance and inflammation by promoting bioconversion of n-3 PUFA and subsequent oxylipin formation. *J Nutr Biochem* **2020**, *76*, 108285, doi:10.1016/j.jnutbio.2019.108285.
20. Cao, H.; Chen, S.F.; Wang, Z.C.; Dong, X.J.; Wang, R.R.; Lin, H.; Wang, Q.; Zhao, X.J. Intervention of 4% salmon phospholipid on metabolic syndrome in mice based on colonic lipidomics analysis. *J Sci Food Agric* **2022**, *102*, 3088-3098, doi:10.1002/jsfa.11649.
21. Moura-Assis, A.; Afonso, M.S.; de Oliveira, V.; Morari, J.; Dos Santos, G.A.; Koike, M.; Lottenberg, A.M.; Ramos Catharino, R.; Velloso, L.A.; Sanchez Ramos da Silva, A.; et al. Flaxseed oil rich in omega-3 protects aorta against inflammation and endoplasmic reticulum stress partially mediated by GPR120 receptor in obese, diabetic and dyslipidemic mice models. *J Nutr Biochem* **2018**, *53*, 9-19, doi:10.1016/j.jnutbio.2017.09.015.
22. Wang, C.; Liu, W.; Yao, L.; Zhang, X.; Zhang, X.; Ye, C.; Jiang, H.; He, J.; Zhu, Y.; Ai, D. Hydroxyeicosapentaenoic acids and epoxyeicosatetraenoic acids attenuate early occurrence of nonalcoholic fatty liver disease. *Br J Pharmacol* **2017**, *174*, 2358-2372, doi:10.1111/bph.13844.
23. Bechynska, K.; Daskova, N.; Vrzackova, N.; Harant, K.; Heczkova, M.; Podzimkova, K.; Bratova, M.; Dankova, H.; Berkova, Z.; Kosek, V.; et al. The effect of omega-3 polyunsaturated fatty acids on the liver lipidome, proteome and bile acid profile: parenteral versus enteral administration. *Sci Rep* **2019**, *9*, 19097, doi:10.1038/s41598-019-54225-8.
24. Skorve, J.; Hilvo, M.; Vihervaara, T.; Burri, L.; Bohov, P.; Tillander, V.; Bjorndal, B.; Suoniemi, M.; Laaksonen, R.; Ekroos, K.; et al. Fish oil and krill oil differentially modify the liver and brain lipidome when fed to mice. *Lipids Health Dis* **2015**, *14*, 88, doi:10.1186/s12944-015-0086-2.

25. Miller, J.L.; Blaszkiewicz, M.; Beaton, C.; Johnson, C.P.; Waible, S., 2nd; Dubois, A.L.; Klemmer, A.; Kiebish, M.; Townsend, K.L. A peroxidized omega-3-enriched polyunsaturated diet leads to adipose and metabolic dysfunction. *J Nutr Biochem* **2019**, *64*, 50-60, doi:10.1016/j.jnutbio.2018.10.010.
26. Rossmeisl, M.; Medrikova, D.; van Schothorst, E.M.; Pavlisova, J.; Kuda, O.; Hensler, M.; Bardova, K.; Flachs, P.; Stankova, B.; Vecka, M.; et al. Omega-3 phospholipids from fish suppress hepatic steatosis by integrated inhibition of biosynthetic pathways in dietary obese mice. *Biochim Biophys Acta* **2014**, *1841*, 267-278, doi:10.1016/j.bbalip.2013.11.010.
27. Daniel, N.; Le Barz, M.; Mitchell, P.L.; Varin, T.V.; Julien, I.B.; Farabos, D.; Pilon, G.; Gauthier, J.; Garofalo, C.; Kang, J.X.; et al. Comparing Transgenic Production to Supplementation of omega-3 PUFA Reveals Distinct But Overlapping Mechanisms Underlying Protection Against Metabolic and Hepatic Disorders. *Function (Oxf)* **2023**, *4*, zqac069, doi:10.1093/function/zqac069.
28. Kubota, T.; Arita, M.; Isobe, Y.; Iwamoto, R.; Goto, T.; Yoshioka, T.; Urabe, D.; Inoue, M.; Arai, H. Eicosapentaenoic acid is converted via omega-3 epoxygenation to the anti-inflammatory metabolite 12-hydroxy-17,18-epoxyeicosatetraenoic acid. *FASEB J* **2014**, *28*, 586-593, doi:10.1096/fj.13-236224.
29. Dong, Y.Q.; Zhang, X.Z.; Sun, L.L.; Zhang, S.Y.; Liu, B.; Liu, H.Y.; Wang, X.; Jiang, C.T. Omega-3 PUFA ameliorates hyperhomocysteinemia-induced hepatic steatosis in mice by inhibiting hepatic ceramide synthesis. *Acta Pharmacol Sin* **2017**, *38*, 1601-1610, doi:10.1038/aps.2017.127.
30. LeMieux, M.J.; Kalupahana, N.S.; Scoggin, S.; Moustaid-Moussa, N. Eicosapentaenoic acid reduces adipocyte hypertrophy and inflammation in diet-induced obese mice in an adiposity-independent manner. *J Nutr* **2015**, *145*, 411-417, doi:10.3945/jn.114.202952.
31. Lu, X.; Huang, L.; Chen, Y.; Hu, L.; Zhong, R.; Chen, L.; Cheng, W.; Zheng, B.; Liang, P. Effect of DHA-Enriched Phospholipids from Fish Roe on Rat Fecal Metabolites: Untargeted Metabolomic Analysis. *Foods* **2023**, *12*, 20, doi:10.3390/foods12081687.
32. Liu, R.; Chen, L.; Wang, Y.; Zhang, G.; Cheng, Y.; Feng, Z.; Bai, X.; Liu, J. High ratio of omega-3/omega-6 polyunsaturated fatty acids targets mTORC1 to prevent high-fat diet-induced metabolic syndrome and mitochondrial dysfunction in mice. *J Nutr Biochem* **2020**, *79*, 108330, doi:10.1016/j.jnutbio.2019.108330.
33. Gao, J.; Xiao, H.; Li, J.; Guo, X.; Cai, W.; Li, D. N-3 Polyunsaturated Fatty Acids Decrease Long-Term Diabetic Risk of Offspring of Gestational Diabetes Rats by Postponing Shortening of Hepatic Telomeres and Modulating Liver Metabolism. *Nutrients* **2019**, *11*, 17, doi:10.3390/nu11071699.
34. Fang, X.; Cai, W.; Cheng, Q.; Ai, D.; Wang, X.; Hammock, B.D.; Zhu, Y.; Zhang, X. Omega-3 PUFA attenuate mice myocardial infarction injury by emerging a protective eicosanoid pattern. *Prostaglandins Other Lipid Mediat* **2018**, *139*, 1-9, doi:10.1016/j.prostaglandins.2018.09.002.
35. Park, J.; Xuan, B.; Jeong, Y.; Han, G.; Kim, E.B. Omega-3-Rich Fish-Oil-Influenced Mouse Gut Microbiome Shaped by Intermittent Consumption of Beef. *Curr Microbiol* **2023**, *80*, 119, doi:10.1007/s00284-023-03223-1.
36. Salsinha, A.S.; Cima, A.; Araujo-Rodrigues, H.; Viana, S.; Reis, F.; Coscueta, E.R.; Rodriguez-Alcala, L.M.; Relvas, J.B.; Pintado, M. The use of an in vitro fecal fermentation model to uncover the beneficial role of omega-3 and punicic acid in gut microbiota alterations induced by a Western diet. *Food Funct* **2024**, *15*, 6095-6117, doi:10.1039/d4fo00727a.
37. Newman, T.M.; Clear, K.Y.J.; Wilson, A.S.; Soto-Pantoja, D.R.; Ochs-Balcom, H.M.; Cook, K.L. Early-life dietary exposures mediate persistent shifts in the gut microbiome and visceral fat metabolism. *Am J Physiol Cell Physiol* **2023**, *324*, C644-C657, doi:10.1152/ajpcell.00380.2021.

38. Zhang, H.; Zheng, L.; Li, C.; Jing, J.; Li, Z.; Sun, S.; Xue, T.; Zhang, K.; Xue, M.; Cao, C.; et al. Effects of gut microbiota on omega-3-mediated ovary and metabolic benefits in polycystic ovary syndrome mice. *J Ovarian Res* **2023**, *16*, 138, doi:10.1186/s13048-023-01227-w.
39. Lu, X.; Zhong, R.; Hu, L.; Huang, L.; Chen, L.; Cheng, W.; Zheng, B.; Liang, P. DHA-enriched phospholipids from large yellow croaker roe regulate lipid metabolic disorders and gut microbiota imbalance in SD rats with a high-fat diet. *Food Funct* **2021**, *12*, 4825-4841, doi:10.1039/d1fo00747e.
40. Zhu, L.; Sha, L.; Li, K.; Wang, Z.; Wang, T.; Li, Y.; Liu, P.; Dong, X.; Dong, Y.; Zhang, X.; et al. Dietary flaxseed oil rich in omega-3 suppresses severity of type 2 diabetes mellitus via anti-inflammation and modulating gut microbiota in rats. *Lipids Health Dis* **2020**, *19*, 20, doi:10.1186/s12944-019-1167-4.
41. Reemst, K.; Tims, S.; Yam, K.Y.; Mischke, M.; Knol, J.; Brul, S.; Schipper, L.; Korosi, A. The Role of the Gut Microbiota in the Effects of Early-Life Stress and Dietary Fatty Acids on Later-Life Central and Metabolic Outcomes in Mice. *mSystems* **2022**, *7*, e0018022, doi:10.1128/msystems.00180-22.
42. Pal, A.; Sun, S.; Armstrong, M.; Manke, J.; Reisdorph, N.; Adams, V.R.; Kennedy, A.; Zu, Y.; Moustaid-Moussa, N.; Carroll, I.; et al. Beneficial effects of eicosapentaenoic acid on the metabolic profile of obese female mice entails upregulation of HEPEs and increased abundance of enteric Akkermansia muciniphila. *Biochim Biophys Acta Mol Cell Biol Lipids* **2022**, *1867*, 159059, doi:10.1016/j.bbalip.2021.159059.
43. Lam, Y.Y.; Ha, C.W.; Hoffmann, J.M.; Oscarsson, J.; Dinudom, A.; Mather, T.J.; Cook, D.I.; Hunt, N.H.; Caterson, I.D.; Holmes, A.J.; et al. Effects of dietary fat profile on gut permeability and microbiota and their relationships with metabolic changes in mice. *Obesity (Silver Spring)* **2015**, *23*, 1429-1439, doi:10.1002/oby.21122.
44. Monk, J.M.; Liddle, D.M.; Hutchinson, A.L.; Wu, W.; Lepp, D.; Ma, D.W.L.; Robinson, L.E.; Power, K.A. Fish oil supplementation to a high-fat diet improves both intestinal health and the systemic obese phenotype. *J Nutr Biochem* **2019**, *72*, 108216, doi:10.1016/j.jnutbio.2019.07.007.
45. Fumagalli, M.; Moltke, I.; Grarup, N.; Racimo, F.; Bjerregaard, P.; Jorgensen, M.E.; Korneliussen, T.S.; Gerbault, P.; Skotte, L.; Linneberg, A.; et al. Greenlandic Inuit show genetic signatures of diet and climate adaptation. *Science* **2015**, *349*, 1343-1347, doi:10.1126/science.aab2319.
46. Tremblay, B.L.; Guenard, F.; Rudkowska, I.; Lemieux, S.; Couture, P.; Vohl, M.C. Epigenetic changes in blood leukocytes following an omega-3 fatty acid supplementation. *Clin Epigenetics* **2017**, *9*, 43, doi:10.1186/s13148-017-0345-3.
47. Bianchi, M.; Alisi, A.; Fabrizi, M.; Vallone, C.; Rava, L.; Giannico, R.; Vernocchi, P.; Signore, F.; Manco, M. Maternal Intake of n-3 Polyunsaturated Fatty Acids During Pregnancy Is Associated With Differential Methylation Profiles in Cord Blood White Cells. *Front Genet* **2019**, *10*, 1050, doi:10.3389/fgene.2019.01050.
48. Massaro, M.; Martinelli, R.; Gatta, V.; Scoditti, E.; Pellegrino, M.; Carluccio, M.A.; Calabriso, N.; Buonomo, T.; Stuppia, L.; Storelli, C.; et al. Transcriptome-based identification of new anti-inflammatory and vasodilating properties of the n-3 fatty acid docosahexaenoic acid in vascular endothelial cell under proinflammatory conditions [corrected]. *PLoS One* **2015**, *10*, e0129652, doi:10.1371/journal.pone.0129652.
49. Bottero, V.; Potashkin, J.A. A Comparison of Gene Expression Changes in the Blood of Individuals Consuming Diets Supplemented with Olives, Nuts or Long-Chain Omega-3 Fatty Acids. *Nutrients* **2020**, *12*, 20, doi:10.3390/nu12123765.

50. Polus, A.; Zapala, B.; Razny, U.; Gielicz, A.; Kiec-Wilk, B.; Malczewska-Malec, M.; Sanak, M.; Childs, C.E.; Calder, P.C.; Dembinska-Kiec, A. Omega-3 fatty acid supplementation influences the whole blood transcriptome in women with obesity, associated with pro-resolving lipid mediator production. *Biochim Biophys Acta* **2016**, *1861*, 1746-1755, doi:10.1016/j.bbalip.2016.08.005.

51. Tait, S.; Calura, E.; Baldassarre, A.; Masotti, A.; Varano, B.; Gessani, S.; Conti, L.; Del Corno, M. Gene and lncRNA Profiling of omega3/omega6 Polyunsaturated Fatty Acid-Exposed Human Visceral Adipocytes Uncovers Different Responses in Healthy Lean, Obese and Colorectal Cancer-Affected Individuals. *Int J Mol Sci* **2024**, *25*, 18, doi:10.3390/ijms25063357.

52. Jimenez-Gomez, Y.; Cruz-Teno, C.; Rangel-Zuniga, O.A.; Peinado, J.R.; Perez-Martinez, P.; Delgado-Lista, J.; Garcia-Rios, A.; Camargo, A.; Vazquez-Martinez, R.; Ortega-Bellido, M.; et al. Effect of dietary fat modification on subcutaneous white adipose tissue insulin sensitivity in patients with metabolic syndrome. *Mol Nutr Food Res* **2014**, *58*, 2177-2188, doi:10.1002/mnfr.201300901.

53. Rangel-Zuniga, O.A.; Camargo, A.; Marin, C.; Pena-Orihuela, P.; Perez-Martinez, P.; Delgado-Lista, J.; Gonzalez-Guardia, L.; Yubero-Serrano, E.M.; Tinahones, F.J.; Malagon, M.M.; et al. Proteome from patients with metabolic syndrome is regulated by quantity and quality of dietary lipids. *BMC Genomics* **2015**, *16*, 509, doi:10.1186/s12864-015-1725-8.

54. Manousopoulou, A.; Scorletti, E.; Smith, D.E.; Teng, J.; Fotopoulos, M.; Roumeliotis, T.I.; Clough, G.F.; Calder, P.C.; Byrne, C.D.; Garbis, S.D. Marine omega-3 fatty acid supplementation in non-alcoholic fatty liver disease: Plasma proteomics in the randomized WELCOME* trial. *Clin Nutr* **2019**, *38*, 1952-1955, doi:10.1016/j.clnu.2018.07.037.

55. Yang, Z.H.; Amar, M.; Sampson, M.; Courville, A.B.; Sorokin, A.V.; Gordon, S.M.; Aponte, A.M.; Stagliano, M.; Playford, M.P.; Fu, Y.P.; et al. Comparison of Omega-3 Eicosapentaenoic Acid Versus Docosahexaenoic Acid-Rich Fish Oil Supplementation on Plasma Lipids and Lipoproteins in Normolipidemic Adults. *Nutrients* **2020**, *12*, 19, doi:10.3390/nu12030749.

56. Okada, L.; Oliveira, C.P.; Stefano, J.T.; Nogueira, M.A.; Silva, I.; Cordeiro, F.B.; Alves, V.A.F.; Torrinhas, R.S.; Carrilho, F.J.; Puri, P.; et al. Omega-3 PUFA modulate lipogenesis, ER stress, and mitochondrial dysfunction markers in NASH - Proteomic and lipidomic insight. *Clin Nutr* **2018**, *37*, 1474-1484, doi:10.1016/j.clnu.2017.08.031.

57. Lambert, C.; Cubedo, J.; Padro, T.; Sanchez-Hernandez, J.; Antonijoan, R.M.; Perez, A.; Badimon, L. Phytosterols and Omega 3 Supplementation Exert Novel Regulatory Effects on Metabolic and Inflammatory Pathways: A Proteomic Study. *Nutrients* **2017**, *9*, 16, doi:10.3390/nu9060599.

58. Bozbas, E.; Zhou, R.; Soyama, S.; Allen-Redpath, K.; Mitchell, J.L.; Fisk, H.L.; Calder, P.C.; Jones, C.; Gibbins, J.M.; Fischer, R.; et al. Dietary n-3 polyunsaturated fatty acids alter the number, fatty acid profile and coagulatory activity of circulating and platelet-derived extracellular vesicles: a randomized, controlled crossover trial. *Am J Clin Nutr* **2024**, *119*, 1175-1186, doi:10.1016/j.ajcnut.2024.03.008.

59. Michielsen, C.; Hangelbroek, R.W.J.; Bragt, M.C.E.; Verheij, E.R.; Wopereis, S.; Mensink, R.P.; Afman, L.A. Comparative Analysis of the Effects of Fish Oil and Fenofibrate on Plasma Metabolomic Profiles in Overweight and Obese Individuals. *Mol Nutr Food Res* **2022**, *66*, e2100192, doi:10.1002/mnfr.202100192.

60. Ding, X.; Xu, Y.; Nie, P.; Zhong, L.; Feng, L.; Guan, Q.; Song, L. Changes in the serum metabolomic profiles of subjects with NAFLD in response to n-3 PUFAs and phytosterol ester: a double-blind randomized controlled trial. *Food Funct* **2022**, *13*, 5189-5201, doi:10.1039/d1fo03921k.

61. Chang, W.C.; So, J.; Lamon-Fava, S. Differential and shared effects of eicosapentaenoic acid and docosahexaenoic acid on serum metabolome in subjects with chronic inflammation. *Sci Rep* **2021**, *11*, 16324, doi:10.1038/s41598-021-95590-7.

62. Xyda, S.E.; Vuckovic, I.; Petterson, X.M.; Dasari, S.; Lalia, A.Z.; Parvizi, M.; Macura, S.I.; Lanza, I.R. Distinct Influence of Omega-3 Fatty Acids on the Plasma Metabolome of Healthy Older Adults. *J Gerontol A Biol Sci Med Sci* **2020**, *75*, 875-884, doi:10.1093/gerona/glz141.

63. Duran, A.M.; Salto, L.M.; Camara, J.; Basu, A.; Paquien, I.; Beeson, W.L.; Firek, A.; Cordero-MacIntyre, Z.; De Leon, M. Effects of omega-3 polyunsaturated fatty-acid supplementation on neuropathic pain symptoms and sphingosine levels in Mexican-Americans with type 2 diabetes. *Diabetes Metab Syndr Obes* **2019**, *12*, 109-120, doi:10.2147/DMSO.S187268.
64. Padro, T.; Lopez-Yerena, A.; Perez, A.; Vilahur, G.; Badimon, L. Dietary omega3 Fatty Acids and Phytosterols in the Modulation of the HDL Lipidome: A Longitudinal Crossover Clinical Study. *Nutrients* **2023**, *15*, 17, doi:10.3390/nu15163637.
65. Smid, V.; Dvorak, K.; Sedivy, P.; Kosek, V.; Lenicek, M.; Dezortova, M.; Hajslova, J.; Hajek, M.; Vitek, L.; Bechynska, K.; et al. Effect of Omega-3 Polyunsaturated Fatty Acids on Lipid Metabolism in Patients With Metabolic Syndrome and NAFLD. *Hepatol Commun* **2022**, *6*, 1336-1349, doi:10.1002/hep4.1906.
66. Yan, M.; Cai, W.B.; Hua, T.; Cheng, Q.; Ai, D.; Jiang, H.F.; Zhang, X. Lipidomics reveals the dynamics of lipid profile altered by omega-3 polyunsaturated fatty acid supplementation in healthy people. *Clin Exp Pharmacol Physiol* **2020**, *47*, 1134-1144, doi:10.1111/1440-1681.13285.
67. Shabrina, A.; Tung, T.H.; Nguyen, N.T.K.; Lee, H.C.; Wu, H.T.; Wang, W.; Huang, S.Y. n-3 PUFA and caloric restriction diet alters lipidomic profiles in obese men with metabolic syndrome: a preliminary open study. *Eur J Nutr* **2020**, *59*, 3103-3112, doi:10.1007/s00394-019-02149-4.
68. Bondia-Pons, I.; Poho, P.; Bozzetto, L.; Vetrani, C.; Patti, L.; Aura, A.M.; Annuzzi, G.; Hyotylainen, T.; Rivellese, A.A.; Oresic, M. Isoenergetic diets differing in their n-3 fatty acid and polyphenol content reflect different plasma and HDL-fraction lipidomic profiles in subjects at high cardiovascular risk. *Mol Nutr Food Res* **2014**, *58*, 1873-1882, doi:10.1002/mnfr.201400155.
69. Ortega, F.J.; Cardona-Alvarado, M.I.; Mercader, J.M.; Moreno-Navarrete, J.M.; Moreno, M.; Sabater, M.; Fuentes-Batllevell, N.; Ramirez-Chavez, E.; Ricart, W.; Molina-Torres, J.; et al. Circulating profiling reveals the effect of a polyunsaturated fatty acid-enriched diet on common microRNAs. *J Nutr Biochem* **2015**, *26*, 1095-1101, doi:10.1016/j.jnutbio.2015.05.001.
70. Veleba, J.; Kopecky, J., Jr.; Janovska, P.; Kuda, O.; Horakova, O.; Malinska, H.; Kazdova, L.; Oliyarnyk, O.; Skop, V.; Trnovska, J.; et al. Combined intervention with pioglitazone and n-3 fatty acids in metformin-treated type 2 diabetic patients: improvement of lipid metabolism. *Nutr Metab (Lond)* **2015**, *12*, 52, doi:10.1186/s12986-015-0047-9.
71. Liu, H.; Li, X.; Zhu, Y.; Huang, Y.; Zhang, Q.; Lin, S.; Fang, C.; Li, L.; Lv, Y.; Mei, W.; et al. Effect of Plant-Derived n-3 Polyunsaturated Fatty Acids on Blood Lipids and Gut Microbiota: A Double-Blind Randomized Controlled Trial. *Front Nutr* **2022**, *9*, 830960, doi:10.3389/fnut.2022.830960.
72. Pu, S.; Khazanehei, H.; Jones, P.J.; Khafipour, E. Interactions between Obesity Status and Dietary Intake of Monounsaturated and Polyunsaturated Oils on Human Gut Microbiome Profiles in the Canola Oil Multicenter Intervention Trial (COMIT). *Front Microbiol* **2016**, *7*, 1612, doi:10.3389/fmicb.2016.01612.
